# Acupuncture for postprandial distress syndrome (APDS): study protocol for a randomized controlled trial

**DOI:** 10.1186/s13063-017-2285-9

**Published:** 2017-11-13

**Authors:** Jing-Wen Yang, Li-Wen Zhang, Guang-Xia Shi, Yi Du, Jun Wang, Jing-Jie Zhao, Yan Cao, Jian-Feng Tu, Shuai Zhang, Cheng Tan, San-San Chen, Cun-Zhi Liu

**Affiliations:** 1 Department of Acupuncture and Moxibustion, Beijing Hospital of Traditional Chinese Medicine affiliated to Capital Medical University, Beijing Key Laboratory of Acupuncture Neuromodulation, No. 23 Meishuguanhou Street, Dongcheng District, Beijing, 100010 China; 2 Department of Traditional Chinese Medicine, Beijing Friendship Hospital, Capital Medical University, Beijing, China; 3 Acupuncture and Moxibustion Department, Dongzhimen Hospital Affiliated to Beijing University of Traditional Chinese Medicine, Beijing, China

**Keywords:** Postprandial distress syndrome, Functional dyspepsia, Acupuncture, Randomized controlled trial, Minimal acupuncture

## Abstract

**Background:**

Postprandial distress syndrome (PDS) is referred to as meal-related functional dyspepsia (FD) and causes a reduced quality of life (QoL) for patients. Several randomized controlled trials (RCTs) have suggested that acupuncture is an effective treatment for FD, but few studies were particularly for PDS. This pilot study was designed to determine the feasibility and efficacy of acupuncture in patients with PDS characterized by postprandial fullness and early satiation according to the Rome III criteria.

**Methods:**

This is a multi-center, two-arm, blinded (participants), pilot RCT. Forty-two participants who meet the inclusion criteria will be randomly assigned to the verum acupuncture group or minimal acupuncture group in a 1:1 ratio. Both treatments consist of 12 sessions of 20 min duration over four weeks (three sessions per week). The primary outcome measurement is the proportion of persons who improve as assessed using the global outcome by the overall treatment effect (OTE) at end-of-treatment (EOT) (four weeks after randomization). Global assessment at weeks 8 and 16 after randomization is one of the secondary outcomes. The other secondary outcomes including symptoms, disease-specific QoL, and depression and anxiety will be assessed at weeks 4, 8, and 16 after randomization.

**Discussion:**

This pilot study will help determine the feasibility and efficacy of acupuncture in patients with PDS.

**Trial registration:**

ISRCTN Registry, ISRCTN18135146. Registered on 7 July 2016.

**Electronic supplementary material:**

The online version of this article (doi:10.1186/s13063-017-2285-9) contains supplementary material, which is available to authorized users.

## Background

Functional dyspepsia (FD) is classified into postprandial distress syndrome (PDS) and epigastric pain syndrome (EPS) based on accumulating epidemiological and pathophysiological data [1, 2]. PDS is a characterized by postprandial fullness and early satiation [2], in the absence of any organic, systemic, or metabolic disease that likely explains the symptoms. Both in the general population and in patients, the most common symptom is postprandial fullness (68–86%)—the main symptom of PDS [3, 4]. PDS might have a higher prevalence of impaired gastric accommodation than EPS [1]. Only a few randomized controlled trials (RCTs) were conducted to examine the treatment responses of PDS and EPS, though testing responses to different FD subgroup are urgently needed [1].

This condition is not a life-threatening disease [5, 6], although patients suffer from a reduced quality of life (QoL) [7–11]. According to a ten-year follow-up study, up to 40% of people with the condition consult a primary care physician [12]. PDS is associated with higher rates of absenteeism and lower productivity at work [13, 14]. Employed people with dyspepsia possibly have a reduced potential productivity of 35.7% [15]. In the United States, costs associated with FD in 2009 were in excess of US$18 billion, meaning that it has substantial financial implications for patients, healthcare organizations, and society [16, 17].

Given the limited efficacy of the major of current treatments for FD, it is not surprising that alternative therapies including acupuncture are attractive to both patients and practitioners [18, 19]. Several RCTs have suggested that acupuncture is an effective treatment for FD [19–21]; however, the studies designed especially for PDS with acupuncture treatment were few. Only a retrospective analysis of acupuncture for FD showed that PDS patients responded better to the acupuncture therapies compared with EPS [22] and acupuncture was not more effective in epigastric pain and epigastric burning (the main symptoms of EPS) than sham acupuncture [23]. The differences in pathological mechanisms of PDS and EPS have been controversial and it remained indistinct whether different treatments are needed for EPS and PDS [1]. Therefore, further RCTs to evaluate the treatment responses of PDS are needed. We designed this study to determine the feasibility and efficacy of acupuncture in patients with PDS.

## Methods/Design

### Study design

**Fig. 1:**
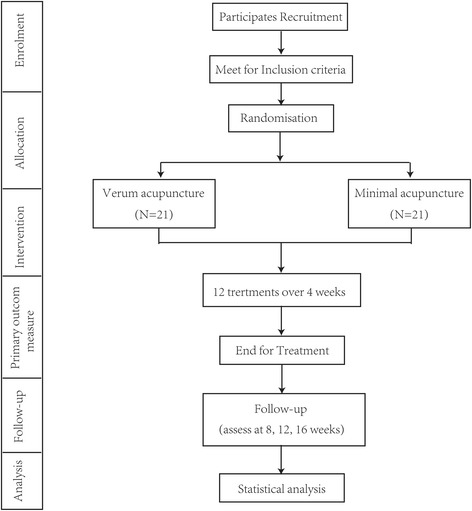
*Flowchart* of trial procedures

Figure 1 shows the study design. This multi-center, two-arm, blinded (participants), pilot RCT will be conducted at three centers: Beijing Hospital of Traditional Chinese Medicine Affiliated to Capital Medical University; Dongzhimen Hospital Affiliated to Beijing University of Chinese Medicine; and Beijing Friendship Hospital Affiliated to Capital Medical University in China. The participants will be recruited primarily through advertisements on hospital social media, general practitioners, and public community service centers. This trial was registered with ISRCTN at Current Controlled Trials (ISRCTN18135146, Additional file 1) and followed the Declaration of Helsinki Good Clinical Practice guidelines for trial conduct. The study protocol (version 1.0, 18 January 2016) has been approved by the Research Ethics Committee of Beijing Hospital of Traditional Chinese Medicine Affiliated to Capital Medical University (reference: 2016BL-011-01) and conformed to CONSORT [24] and STRICTA guidelines [25] for acupuncture studies (Additional file 2). Before randomization, all participants will be requested to provide written informed consent.

### Eligibility

#### Inclusion criteria

1. Aged 18–65 years (either sex)
2. Meeting the Rome III PDS criteria
3. Normal esophagogastroduodenoscopy results within one year
4. No other treatments received during the study
5. Willing to sign written informed consent

#### Exclusion criteria

1. Presence of serious structural disease (disease of heart, lung, liver, or kidney)
2. Signs of irritable bowel syndrome
3. Surgery related with the gastrointestinal tract
4. Severe coagulopathy
5. Taking drugs that might affect dyspepsia, such as anti-secretary drugs, antacids, prokinetics, non-steroidal anti-inflammatory drugs, and antidepressants two weeks before participating in the trial
6. Difficulties in attending the trial, such as serious mental illness, dementia, or illiteracy
7. Drug or alcohol abuse
8. Pregnant or breastfeeding

#### Randomization and allocation concealment

All participants who meet the inclusion criteria will be randomly assigned to the verum acupuncture group or minimal acupuncture group in a 1:1 ratio according to a randomization sequence generated using SPSS19.0 software. The randomization sequence will be prepared by a professional statistician, who will not be involved in assessment or treatment of participants. An independent clinical trials researcher will implement the allocation schedule using a centralized telephone randomization procedure. The random number will be assigned after the participants have met all inclusion criteria and completed baseline assessment. The Clinical Research Coordinator (CRC) will be responsible for enrolling participants, obtaining informed consent and requesting randomization.

#### Blinding

The acupuncturists who deliver the treatment to the participants will not be blind to treatment allocation. Blinding of acupuncturists is not possible due to the nature of acupuncture. Participants and all other investigators will be blinded to the treatment allocation, including the data analysis and outcome assessors. Treatment groups will be identified as Group A and Group B during the statistical analysis of all the data for data analysis.

#### Intervention

The treatment protocol for acupuncture was developed using the clinical experience of acupuncture experts for reference. Verum acupuncture and minimal acupuncture will be performed by certified acupuncturists who hold a Chinese medicine practitioner license from the Ministry of Health of the People’s Republic of China and worked for at least three years in clinics. Before the trial, all the acupuncturists will be required to take special training to acquire a full understanding of the performance of treatment and receive a brochure showing detailed information on the standardized operation. The training included the method of the location of acupoints and non-acupoints as well as manipulation of acupuncture and minimal acupuncture. We will use sterile needles (Huatuo disposable acupuncture needle) in sizes of 0.25 × 25 mm or 0.25 × 40 mm. Both the verum acupuncture and minimal acupuncture treatments consist of 12 sessions of 20 min duration over four weeks (three sessions per week). The use of other treatments related to PDS, such as prokinetic agents or tricyclic antidepressants, will not be allowed.

#### Verum acupuncture

**Fig. 2:**
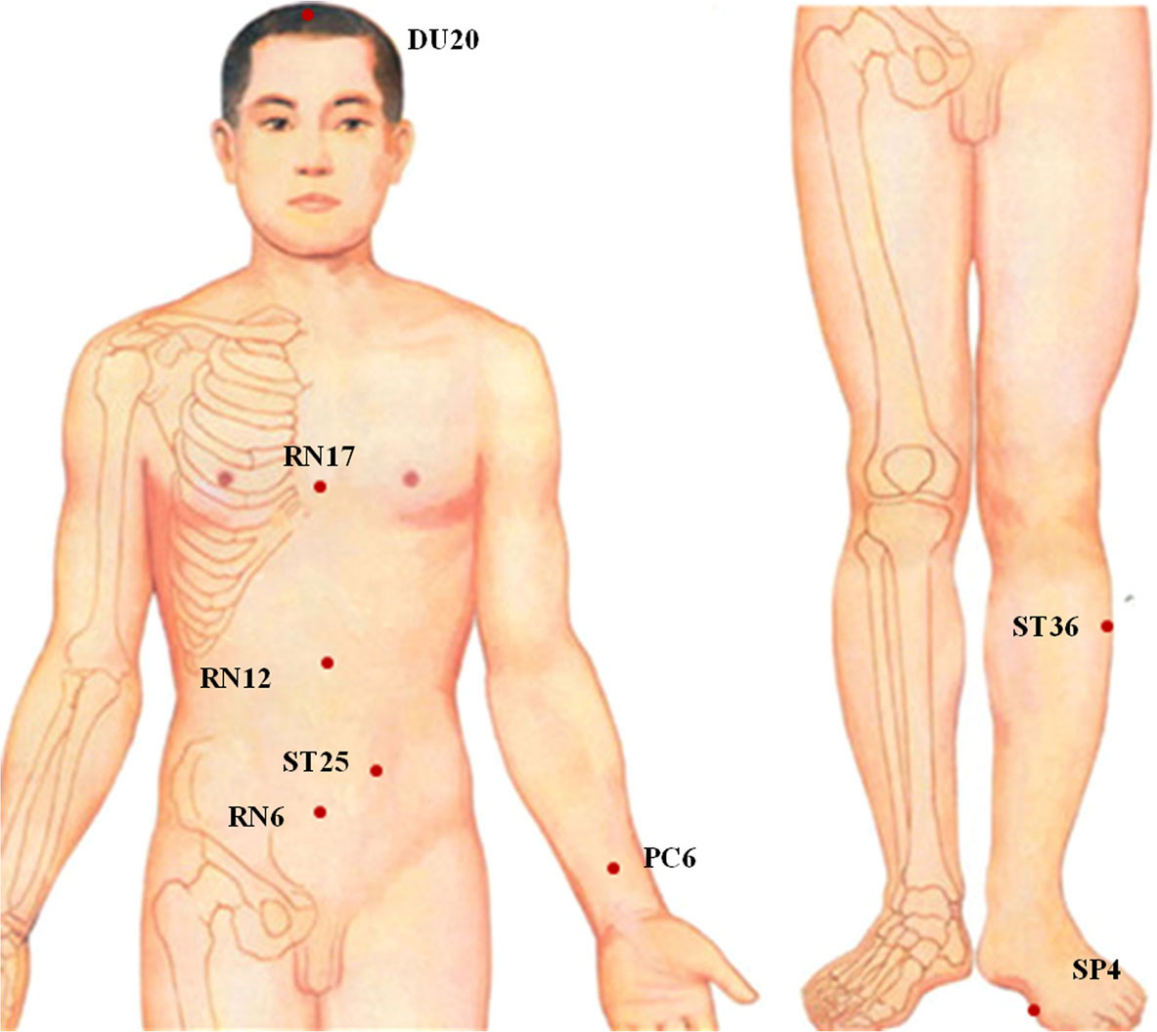
Location of acupoints in verum acupuncture group

Participants randomized to the verum acupuncture group will undergo treatment lying down with needles inserted at the selected acupuncture points. Table 1 and Fig. 2 describe the verum acupuncture point prescription. Acupuncturists will be required to achieve the typical acupuncture sensation of De qi and needles will be stimulated manually for at least 30 s at every acupoint and retained in place for 20 min. De qi, literally meaning “arrival of energy,” is referred to as a sensation of numbness or distension and may be one indication that acupuncture is exerting its beneficial effects.

**Table 1:** Location of acupoints in verum acupuncture group

Acupoints	Location
Baihui (DU20)	On the midline of the head, 7 cun^a^ directly above the midpoint of the posterior hairline
Zhongwan (RN12)	On the anterior midline, 4 cun above the umbilicus
Tianshu (ST25)	On the same level of the umbilicus and 2 cun lateral to the anterior midline
Qihai (RN6)	On the anterior midline, 1.5 cun below the umbilicus
Neiguan (PC6)	On the line joining Daling and Quze, between the tendons of palmaris longus and flexor carpi radialis, 2 cun above the transverse crease of the wrist
Danzhong (RN17)	On the anterior midline, on the level of the fourth intercostal space, at the midpoint of the line joining the two nipples
Zusanli (ST36)	3 cun directly below Dubi (ST35) and one finger-breadth lateral to the anterior border of the tibia
Gongsun (SP4)	On the medial side of the foot, in the depression anterior and inferior to the first metatarsal bone, at the junction of the red and white skin

^a^One “cun” is defined as the width of the interphalangeal joint of patient’s thumb

#### Minimal acupuncture

**Fig. 3:**
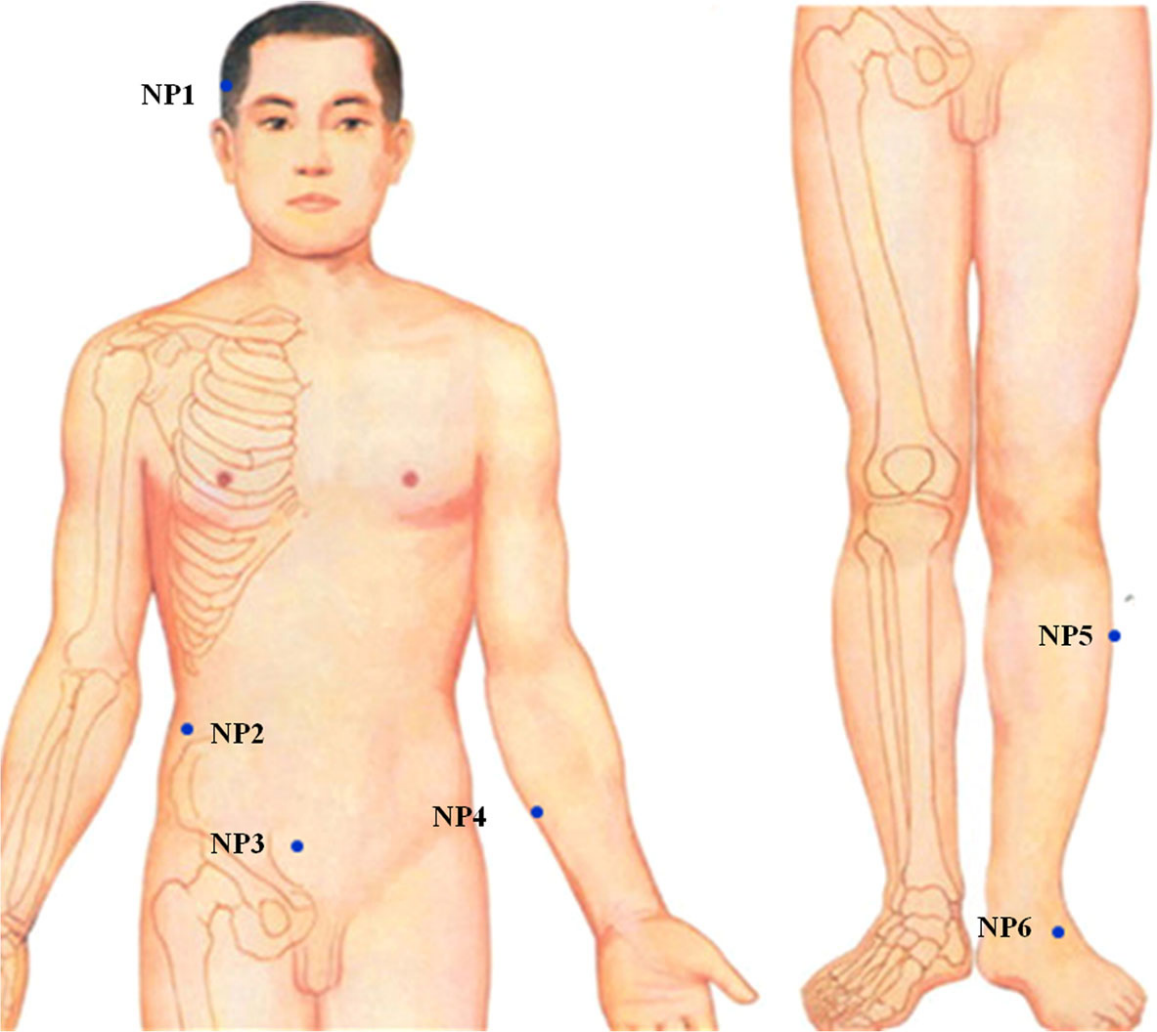
Location of non-acupoints in minimal acupuncture group

Non-acupoints with a superficial puncture (2 mm in depth) will be performed in minimal acupuncture group, without considering a needle sensation and manual stimulation. The locations of non-acupoints are away from any acupoints or meridians and shown in Table 2 and Fig. 3. The treatment disposal will be the same as in the verum acupuncture group.

**Table 2:** Location of non-acupoints in minimal acupuncture group

Non-acupoints	Location
NP1	In the middle of Jiaosun (SJ20) and Shuaigu (GB8) points
NP2	2.0 cun^a^ above the anterior superior iliac spine
NP3	2.0 cun below the umbilicus and 1.0 cun lateral to the anterior midline
NP4	In the middle of the medial epicondyle of the humerus and the styloid process of ulna
NP5	3.0 cun below Yanglingquan (GB34), between the gallbladder and bladder meridian
NP6	In the middle of Qiuxu (GB40) and Jiexi (ST41) points

^a^One “cun” is defined as the width of the interphalangeal joint of patient’s thumb

#### Outcomes

Table 3 summarizes the outcomes and the time-points of data collection during the trial.

**Table 3:** Time to visit and data collection

Outcomes	Baseline	Treatment				Follow-up		
		1 weeks	2 weeks	3 weeks	4 weeks	8 weeks	12 weeks	16 weeks
Patients								
Informed consent	×							
Screening	×							
Sign informed consent	×							
Randomization	×							
Primary outcome								
OTE					×			
Second outcome								
OTE		×	×	×		×	×	×
Symptoms	×	×	×	×	×	×	×	×
NPI	×				×	×		×
HADS	×				×	×		×
Adverse events		×	×	×	×			

*OTE* overall treatment effect, *NPI* Nepean Dyspepsia Index, *HADS* Hospital Anxiety Depression Scale

#### Primary outcome measurement

The primary outcome is the responder rate based on the overall treatment effect (OTE) at end-of-treatment (EOT, four weeks after randomization) [26, 27]. The patient will be asked to decide whether symptoms have changed compared with pre-treatment using a Likert scale. The question will be asked “How were your gastric symptoms during the past week in comparison with the baseline period?” The seven-point Likert scale consists of “extremely improved,” “improved,” “slightly improved,” “not changed,” “slightly aggravated,” “aggravated,” and “extremely aggravated” [27]. Patients who are “extremely improved” or “improved” of the OTE will be considered responders. OTE has been used as the primary and secondary outcomes measure in several trials with FD [27–31]. The advantage of this endpoint is that it closely resembles the way physicians evaluate treatment benefit in clinical practice, but the main disadvantage is the inherent recall of pre-treatment symptom severity which may lead to bias [26].

### Secondary outcome measurements

#### Global assessment at other time-points

The primary outcome measurement is the OTE at the EOT; however, responder rate based on OTE will also be measured weekly (during the treatment) and at weeks 8, 12, and 16 after randomization with a seven-point Likert scale [27].

#### Symptoms assessment

The eight dyspepsia symptoms including postprandial fullness, early satiation, upper abdominal bloating, epigastric pain, epigastric burning, nausea, vomit, and belching will be assessed at baseline, once a week for four weeks in the treatment period and at weeks 8, 12, and 16 after randomization. The severity of each symptom is rated as a four-item questionnaire: asymptomatic (0 points); mild (1 point); moderate (2 points); or severe (3 points) [20]. A higher score reflects a more serious disease state.

#### Disease-specific QoL assessment

QoL will be assessed at baseline and at weeks 4, 8, and 16 after randomization using the 25-item Nepean Dyspepsia Index (NDI) [32–34], which measures the specific life quality of FD patients in four domains: interference (13 items); know/control (seven items); eat/drink (three items); and sleep/disturb (two items). The scores are measured using a five-point Likert scale that ranges from “not at all” to “extremely,” with higher scores indicating a better QoL.

#### Depression and anxiety assessment

Depression and anxiety symptoms will be assessed by the Hospital Anxiety Depression Scale (HADS), which has been validated for in a wide variety of general medical patients. It is a self-report inventory consisting of 14 questions, seven of which relate to anxiety symptoms and seven to depressive symptoms. A higher score reflects a more serious state of depression and anxiety. The participants will complete the HADS at baseline and at weeks 4, 8, and 16 after randomization.

#### Adverse events

Any adverse events related to acupuncture will be monitored and recorded by participants or acupuncturists using a designed questionnaire. Possible adverse events are local bleeding, redness, itching at the sites of needle insertion, and dizziness throughout treatment. Adverse events will be assessed by outcome assessors weekly during the treatment period.

#### Data management

All researchers including therapists, data collector, data entry clerks, data manager, statistician, and outcome assessors will receive training regarding the data management. Upon conclusion of the treatment period, all participant data will be completed and recorded on the original case report forms (CRFs). The data will be entered into Excel spreadsheets by two separate data entry clerks, following which the data manager will compare the accuracy of the two datasets. If any differences are noted, corrections will be made according to the original CRFs.

All paper files related to the research will be saved and electronic documents will be stored in a password-protected computer. All research documents, including both the paper files and electronic documents, will be preserved for at least five years after publication. If readers have any questions regarding our published data, they will be permitted to contact our first author or corresponding author to ask for the original data. The private information of patients including name, age, and telephone number, will be protected and never disclosed to anyone.

In addition, we will establish an independent Data and Safety Monitoring Board (DSMB) to review and interpret data generated from the study (Additional file 3). The primary objective of DSMB is to ensure the integrity of the research data. The DSMB will review the progress of the trial and decide on any premature closure of the study.

#### Sample size

This pilot study aims to assess the efficacy of acupuncture for PDS as well as the feasibility of a further large clinical trial. We did not perform a sample size calculation for this pilot study, but will use a convenience sample based on the known availability of study participants at the three sites. We estimate that we will be able to enroll 42 individuals, 14 in each center, for this pilot study. Both the verum acupuncture and minimal acupuncture groups will contain 21 eligible participants. The outcomes of this study will facilitate the calculation of the appropriate sample size for further RCTs.

#### Statistical analysis

The statistical analysis will be performed by an independent statistician who is blinded to group allocation using SPSS 19.0 (IBM SPSS Statistics, New York, NY, USA). All efficacy analyses will be performed using the intent-to-treat (ITT) and per-protocol (PP) population. For ITT analysis, the population will consist of all participants who have been randomized and received acupuncture treatment at least once and the last observation carried forward rule will be applied. The PP analysis will include only those participants who will complete more than ten acupuncture treatments and have no major protocol violations (taking other drugs during the trial, not completing the CRF as required, etc.). The categorical data will be described as percentage (n %) and continuous data using mean ± standard deviations. Continuous variables will be analyzed using the t-test and categorical variables using the Chi-square (χ^2^) test. The statistical significance level will be set at 0.05 (two-sided) with 95% confidence intervals.

## Discussion

People with PDS have a normal life expectancy, but the impact on QoL is notable. This pilot study has been designed, therefore, to evaluate whether acupuncture will improve the symptoms of PDS (postprandial fullness and early satiation) and be a feasible therapy for clinical treatment.

Whether different treatments are needed for EPS and PDS is indistinct and in the previous RCTs, only acotiamide, a new drug for the treatment of FD, has been certified to be effective in PDS but not in EPS [30]. We are the first RCT designed for PDS with acupuncture treatment. Our study meets methodological theory of adequate randomization and allocation concealment, blinding of outcome assessors and statisticians, and applying ITT analysis strategy. In addition to PDS symptoms and disease-specific QoL, the depression and anxiety status of the patients will also be measured in our study.

The primarily limitation of our trial is that acupuncturists could not be blinded, but we provided special training and provide a brochure to minimize bias. Second, in consideration of the ethical requirements and patient compliance, a blank control group was not designed and the influence of self-healing could not be excluded from the acupuncture effect. This pilot RCT aims to evaluate the feasibility and efficacy of acupuncture for PDS and, if effective, a further large clinical will be conducted.

### Trial status

This trial is currently recruiting participants.

## Additional files

Additional file 1: Trial registration: Acupuncture for postprandial distress syndrome (PDS). (PDF 23 kb)

Additional file 2: SPIRIT 2013 Checklist: Recommended items to address in a clinical trial protocol and related documents.* (PDF 192 kb)

Additional file 3: DSMB of Acupuncture for postprandial distress syndrome. (JPG 640 kb)

## Abbreviations

EOT: End-of-treatment
EPS: Epigastric pain syndrome
FD: Functional dyspepsia
HADS: Hospital Anxiety Depression Scale
ITT: Intent-to-treat
NDI: Nepean Dyspepsia Index
OTE: Overall treatment effect
PDS: Postprandial distress syndrome
PP: Per-protocol
QoL: Quality of life
