# Supplementary material for: Acupuncture for postprandial distress syndrome (APDS): study protocol for a randomized controlled trial
Source: Trials. 2017 Nov 13;18:537. doi: 10.1186/s13063-017-2285-9 (PMC5683366; doi:10.1186/s13063-017-2285-9)
Supplement: Supplementary file 1 — Trial registration: Acupuncture for postprandial distress syndrome (PDS). (PDF 23 kb) [file 13063_2017_2285_MOESM1_ESM.pdf]

ISRCTN18135146 DOI 10.1186/ISRCTN18135146

Acupuncture for postprandial distress syndrome (PDS)

Plain English Summary

Background and study aims
Functional dyspepsia (FD) is a long-term condition of the digestive system, that causes discomfort in the upper belly, near the ribs. Although FD is not a life-threatening disease, it has considerable impact on quality of life. There are various available drug treatments for FD which neutralize stomach acid or stop the stomach from producing so much, but many FD patients turn to complementary and alternative therapies as often medications do not provide enough relief. Acupuncture is a popular alternative therapy, derived from ancient Chinese medicine. Although it has been frequently used to treat symptoms of FD, evidence of its effectiveness is lacking. The Rome III consensus subdivided FD into two subgroups: postprandial distress syndrome (an unpleasant feeling of fullness after eating) and epigastric pain syndrome (upper abdominal pain and burning). However, few studies have been conducted to examine the treatment responses of different FD subgroups. The aim of this study is to assess the efficacy of acupuncture in the treatment of postprandial distress syndrome (PDS) patients.

Who can participate?
Male and female patients aged 18-65 diagnosed with PDS.

What does the study involve?
Patients are randomly allocated to one of two groups: a verum acupuncture group or a minimal acupuncture group. The verum acupuncture sessions involve placing needles in specific points on the body, which are stimulated by hand for at least 30 seconds to achieve the typical acupuncture sensation. The minimal acupuncture sessions involve the placement of needles in non-acupoints (places not intended to cause an effect). Both verum acupuncture and minimal acupuncture treatments consist of 12 sessions of 20 minutes duration over 4 weeks (three sessions per week). Participants in both groups are followed up 4, 8 and 16 weeks after first acupuncture.

What are the possible benefits and risks of participating?

Participants may benefit from an improvement to their symptoms and general quality of life. The risks of receiving acupuncture are minimal. Acupuncture is a relatively safe treatment when given by properly trained clinicians. Occasionally, acupuncture can make people feel nauseous or faint. Participants are warned of these potential side-effects before receiving acupuncture.

Where is the study run from?
1. Beijing Hospital of Traditional Chinese Medicine affiliated to Capital Medical University (China)
2. Dongzhimen Hospital Affiliated to Beijing University of Chinese Medicine (China)
3. Beijing Friendship Hospital Affiliated to the Capital Medical University (China)

When is the study starting and how long is it expected to run for?
March 2016 to January 2017

Who is funding the study?
Beijing Municipal Science & Technology Commission (China)

Who is the main contact?
Dr Liu Cun-Zhi

Trial website

Contact information

Type

Scientific

Primary contact

Mr Cun-Zhi Liu

ORCID ID

Contact details

Beijing Hospital of Traditional Chinese Medicine affiliated to Capital Medical University
23 Meishuguanhou Street
Dongcheng District
Beijing

100010
China
Additional identifiers
EudraCT number
ClinicalTrials.gov number
Protocol/serial number
Z161100000516007
Study information
Scientific title
Acupuncture for postprandial distress syndrome (APDS): a pilot randomized controlled trial
Acronym
APDS
Study hypothesis
The efficacy of verum acupuncture on postprandial distress syndrome (PDS) is superior to minimal acupuncture.
Ethics approval
Research Ethical Committee of Beijing Hospital of Traditional Chinese Medicine affiliated to Capital Medical University, 14/03/2016, ref: 2016BL-011-01
Study design
Two-arm multi-centre randomized controlled pilot trial
Primary study design
Interventional
Secondary study design
Randomised controlled trial
Trial setting
Hospitals
Trial type
Treatment
Patient information sheet
No specific participant information sheet available, please use the contact details below to request a further information.
Condition
Postprandial distress syndrome (PDS)

Intervention

Participants will be randomly assigned to verum acupuncture at acupoints or minimal acupuncture at non-acupoints in a 1:1 ration

Verum acupuncture group: Participants in the verum acupuncture group will receive a standardized 20-minute acupuncture session needling at selected points: Baihui(DU20), zhongwan(RN12), Tianshu(ST25), Qihai(RN6), Neiguan(PC6), Danzhong(RN17), Zusanli(ST36), Gongsun(SP4). Needles will be stimulated manually at least 30 seconds to achieve the typical acupuncture sensation (deqi). Treatments consist of 12 sessions of 20 minutes duration over 4 weeks (three sessions per week).

Minimal acupuncture group: Participants in the minimal acupuncture group will receive 20-minute acupuncture at non-acupoints. Needles will be placed at non-acupoints with a superficial puncture (2 mm in depth) to avoid de qi and manual stimulation. The location of non-acupoints as following:

1. In the middle of Jiaosun(SJ20) and Shuaigu(GB8) points
2. 2.0 cun above the anterior superior iliac spine
3. 2.0 cun below the umbilicus, and 1.0 cun lateral to the anterior midline
4. In the middle of the medial epicondyle of the humerus and the styloid process of ulna
5. 3.0 cun below Yanglingquan(GB34), between the gallbladder and bladder meridian
6. In the middle of Qiuxu(GB40) and Jiexi(ST41) points

Treatments consist of 12 sessions of 20 minutes duration over 4 weeks (three sessions per week).

The use of other treatments related to PDS will not be allowed for participans in either group. The study will include 4 weeks treatment and 12 weeks follow-up.

Intervention type

Phase

Drug names

Primary outcome measures

Overall treatment effect (OTE) is measured using patient interviews using a Likert scale at baseline, once a week for the four week treatment period and then at 4, 8 and 16 weeks after first acupuncture.

Secondary outcome measures

1. Symptoms and global assessment of PDS patients are measured using a four-item questionnaire-asymptomatic (0 point), mild (1 point), moderate (2 points) or severe (3 points)-including eight symptoms: postprandial distension, early satiety, epigastric pain, epigastric burning, upper abdominal bloating, nausea. Assessment will be conducted at baseline, twice a week in treatment period and 8, 16 weeks after first acupuncture.
2. Quality of life is evaluated using the 25-item Nepean Dyspepsia Index (NDI) at baseline, 4, 8 and 16 weeks after first acupuncture
3. Severity of anxiety and depression will be graded using the Hospital Anxiety Depression Scale (HADS) at baseline, 4, 8 and 16 weeks after first acupuncture

Overall trial start date

01/03/2016

Overall trial end date

31/01/2017

Reason abandoned

Eligibility

Participant inclusion criteria

1. Aged between 18 and 65 years
2. Normal esophagogastroduodenoscopy results within a year
3. Not allowed drug: anti-secretory drugs, antacids, prokinetics, non-steroidal anti-inflammatory drugs and antidepressant drugs during the treatment period
4. No other treatments received during the study
5. Willing to sign written informed consent

Participant type

Patient

Age group

Adult

Gender

Both

Target number of participants

42

Participant exclusion criteria

1. Presence of serious structural disease(disease of heart, lung, liver or kidney), malignant or mental disease
2. Signs of irritable bowel syndrome
3. Surgery related with the gastrointestinal tract
4. Severe coagulopathy
5. Drug or alcohol abuse
6. Pregnant or breastfeeding

Recruitment start date

30/06/2016

Recruitment end date

31/10/2016

Locations

Countries of recruitment

China

Trial participating centre

Beijing Hospital of Traditional Chinese Medicine affiliated to Capital Medical University

Beijing

100010

China

Trial participating centre

Dongzhimen Hospital Affiliated to Beijing University of Chinese Medicine

Beijing

100700

China

Trial participating centre

Beijing Friendship Hospital Affiliated to the Capital Medical University

Beijing

100069

Sponsor information

Organisation

Beijing Municipal Science & Technology Commission

Sponsor details

Building 2

No. 7

Evergreen Road

Haidian District

Beijing

100195

China

Sponsor type

Government

Website

Funders

Funder type

Government

Funder name

Beijing Municipal Science & Technology Commission

Alternative name(s)

Funding Body Type

Funding Body Subtype

Location

Results and Publications

Publication and dissemination plan

Planned publication in a peer reviewed journal.

Intention to publish date

31/03/2017

Participant level data

Not expected to be available

Results - basic reporting

Publication summary

Publication citations

Additional files

Editorial Notes
