# Supplementary figures and images for: Acupuncture for postprandial distress syndrome (APDS): study protocol for a randomized controlled trial
Source: Trials. 2017 Nov 13;18:537. doi: 10.1186/s13063-017-2285-9 (PMC5683366; doi:10.1186/s13063-017-2285-9)

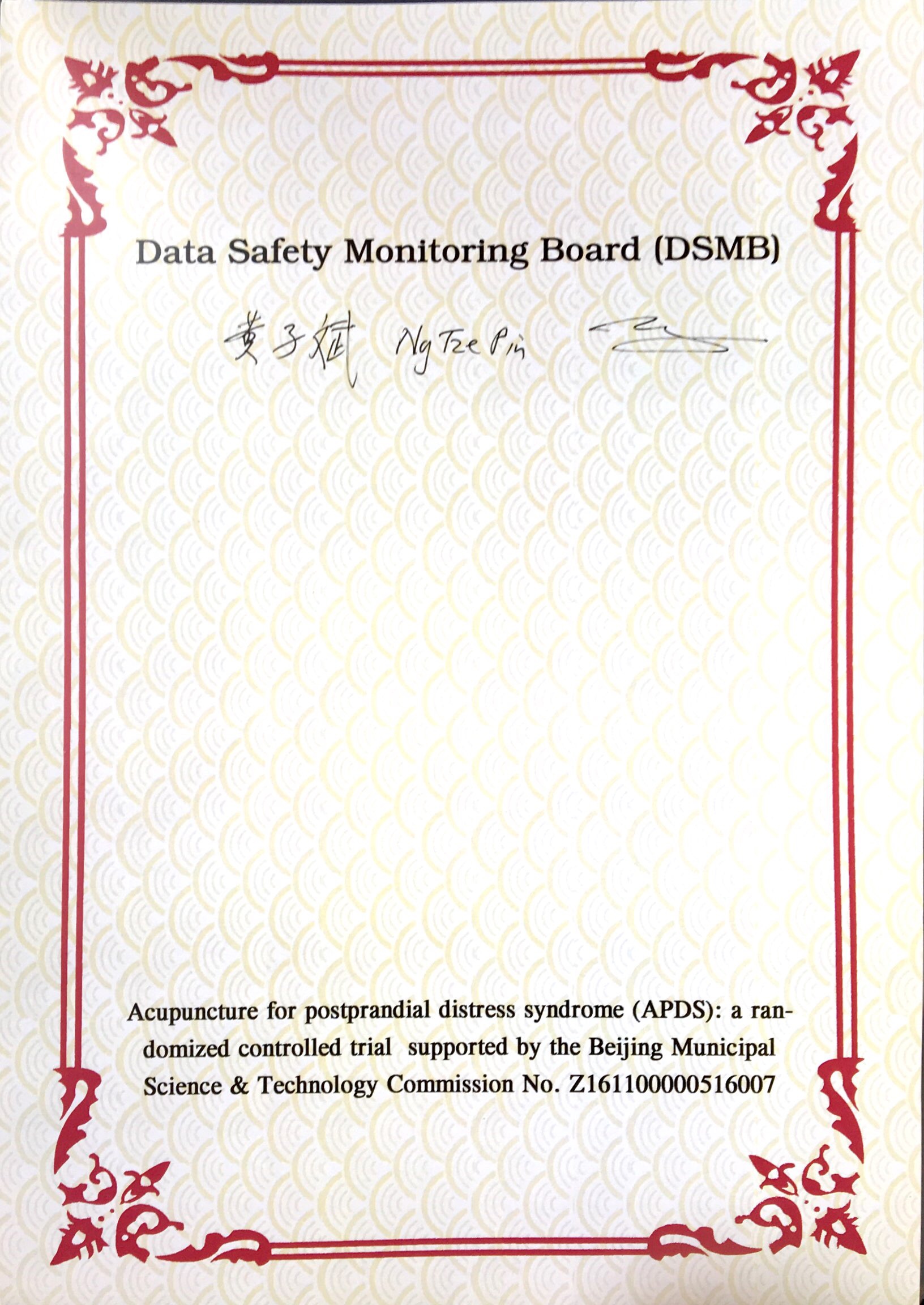

Supplement: Supplementary file 3 — DSMB of Acupuncture for postprandial distress syndrome. (JPG 640 kb) [file 13063_2017_2285_MOESM3_ESM.jpg]
